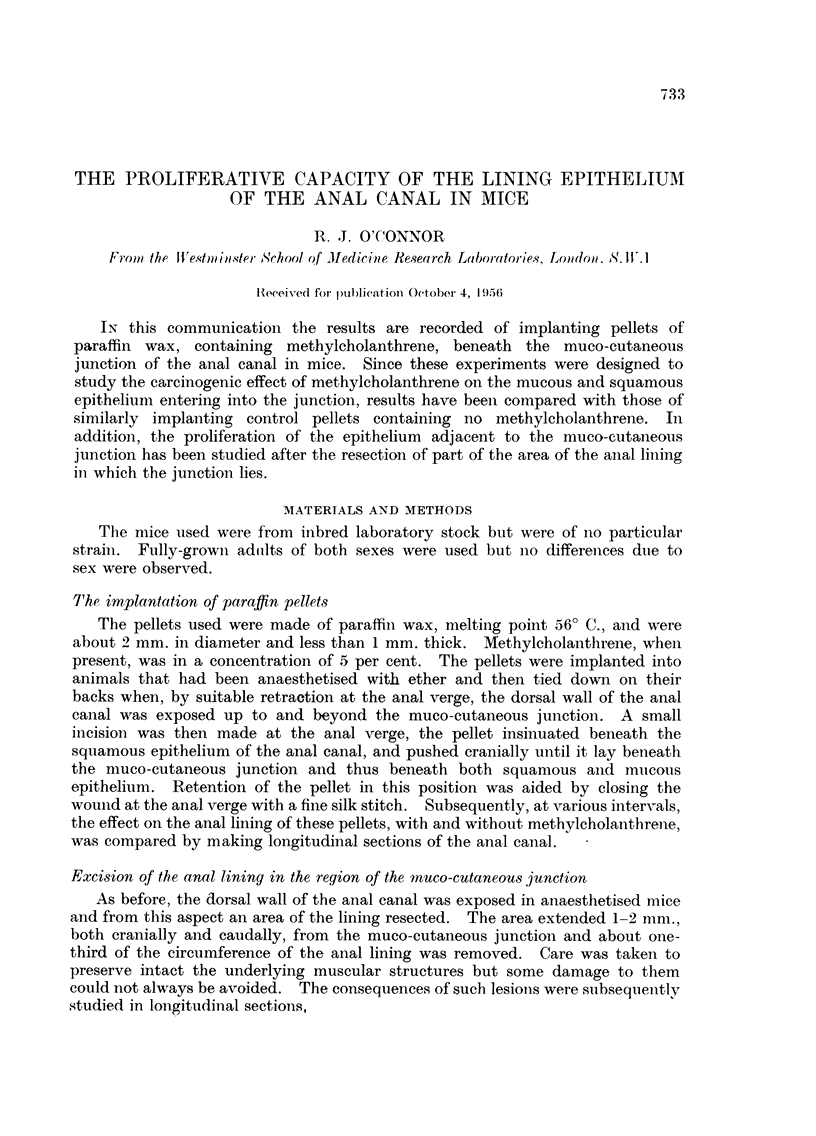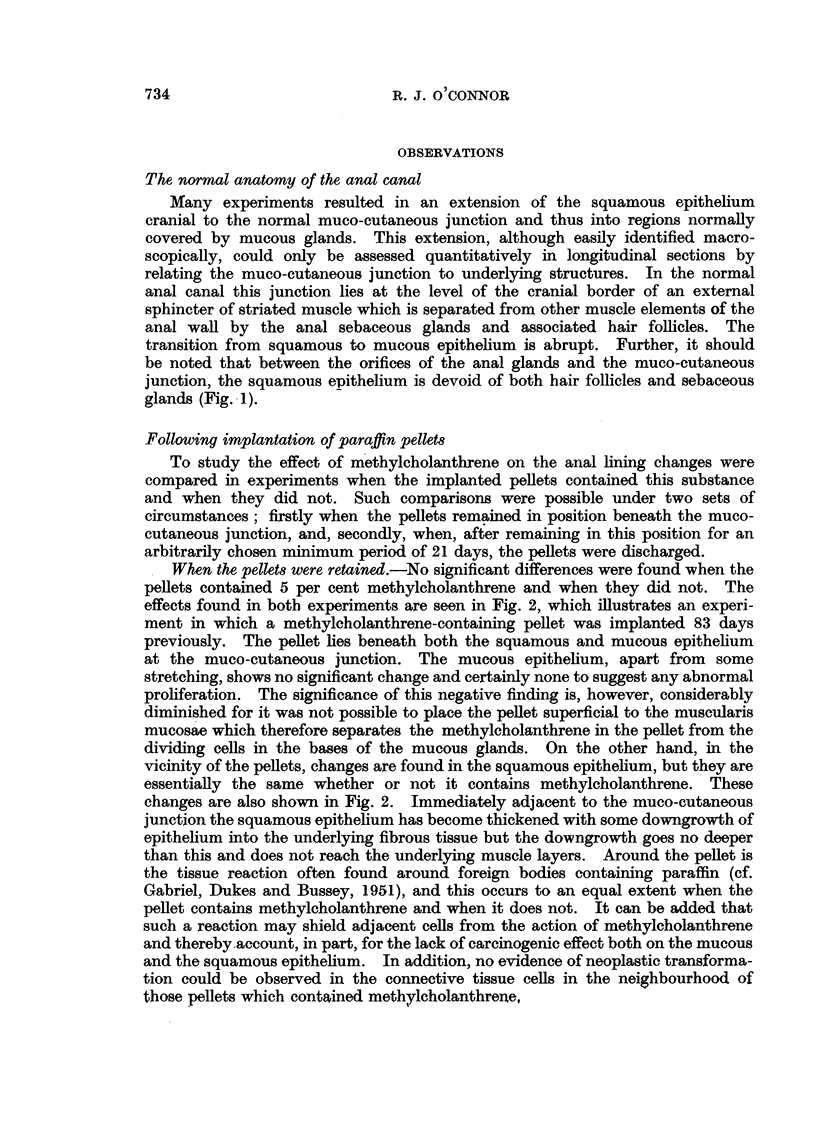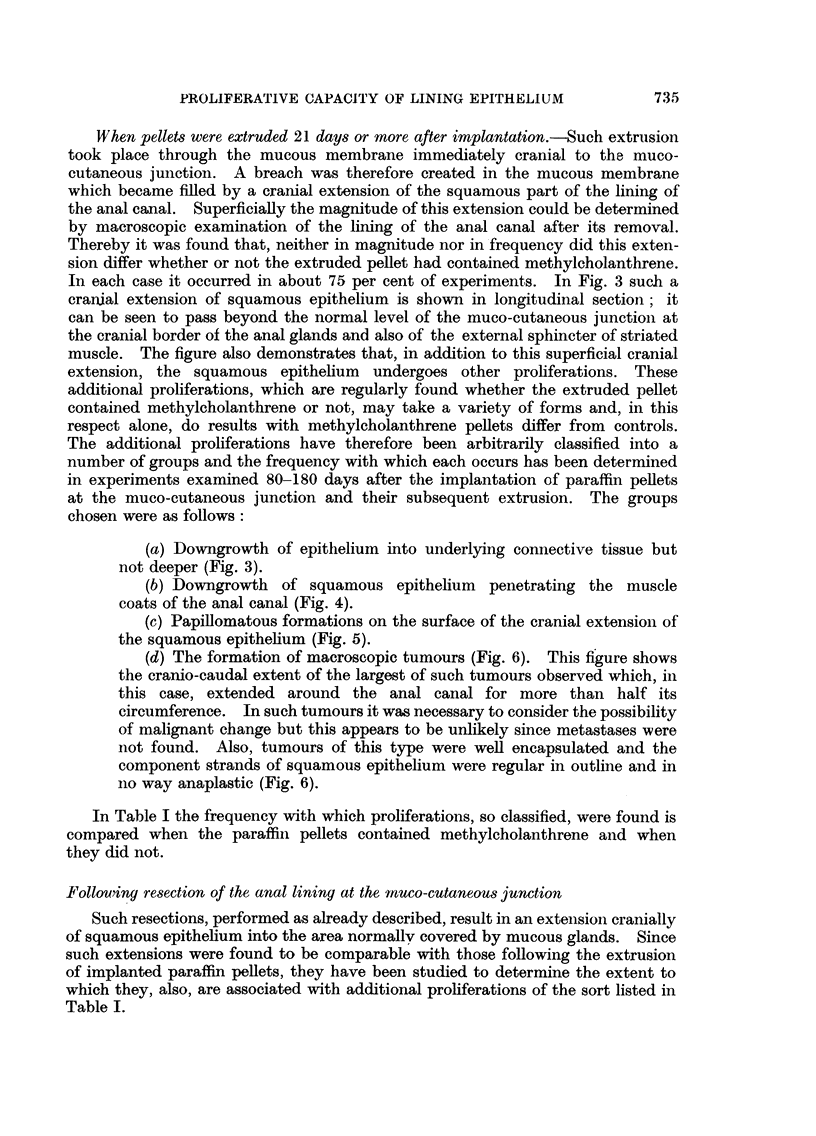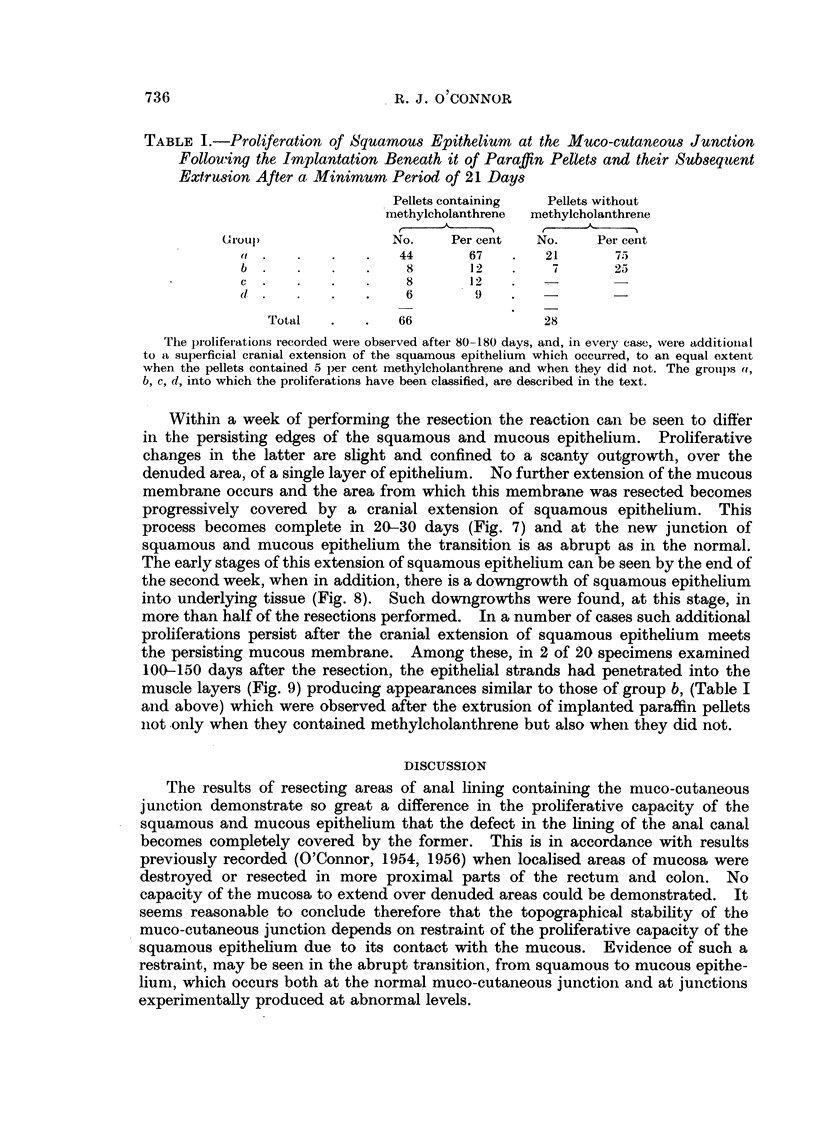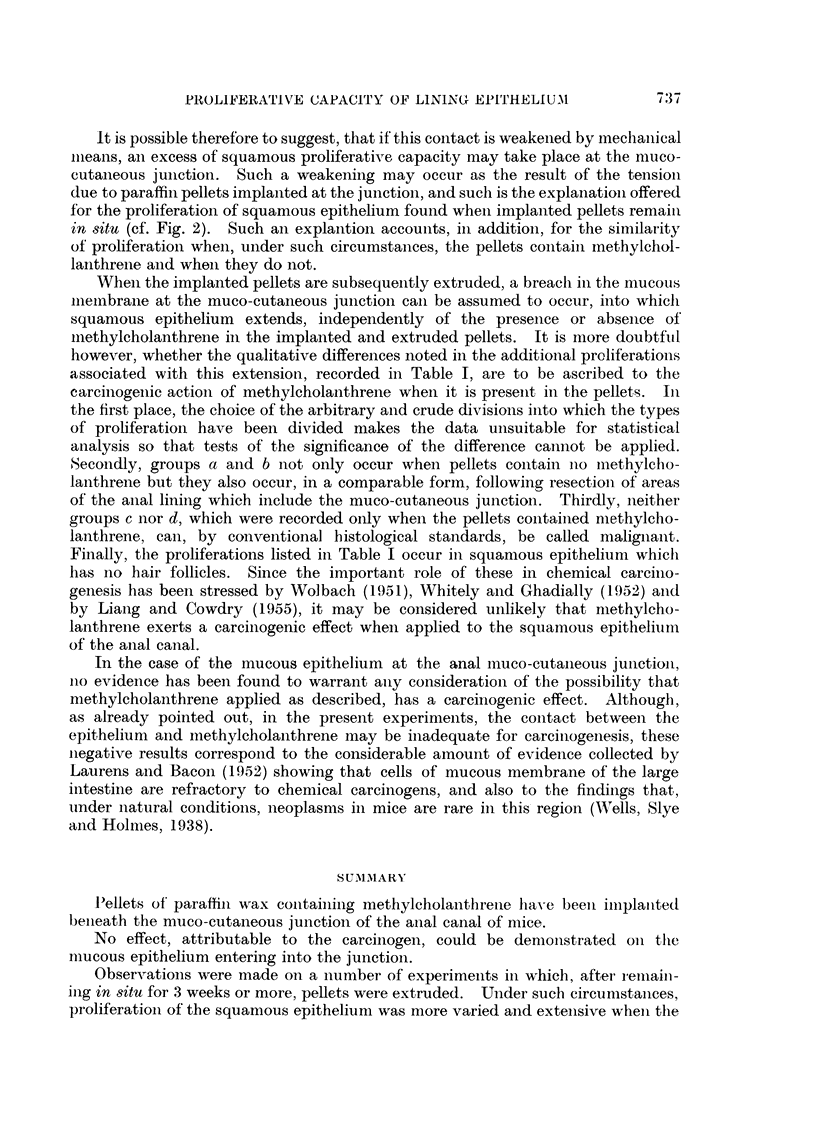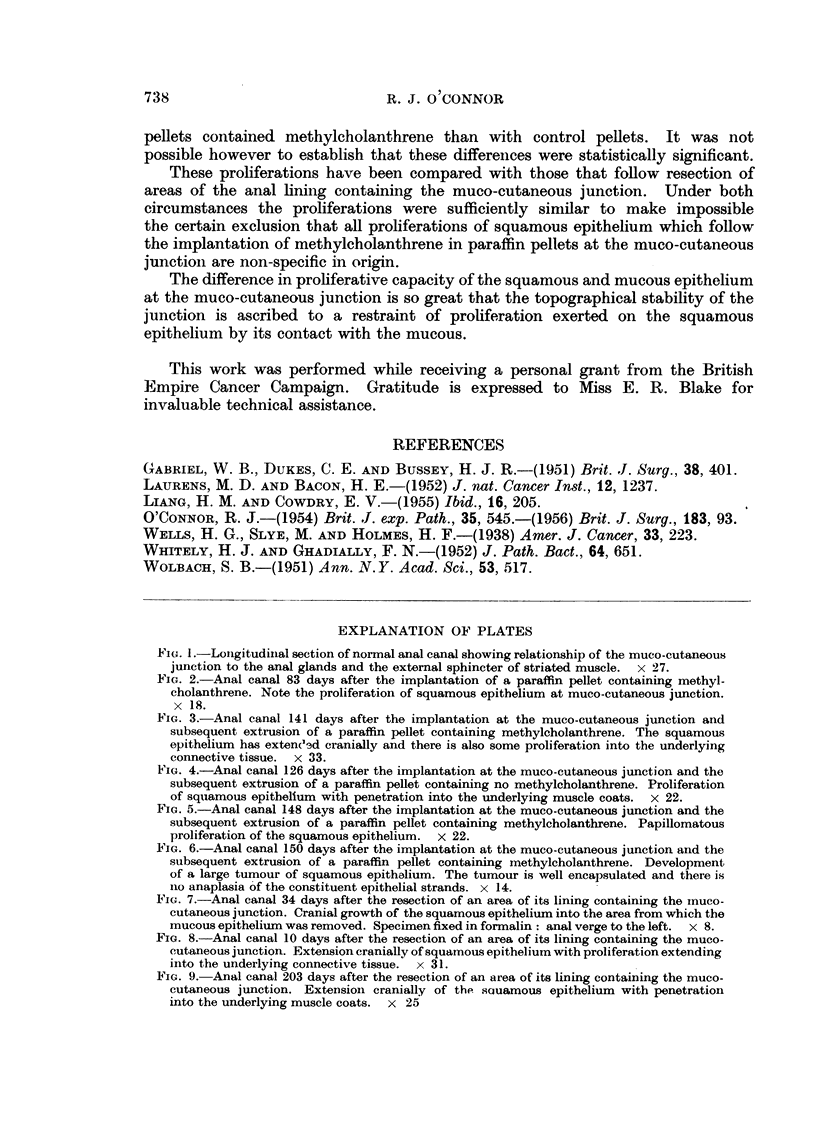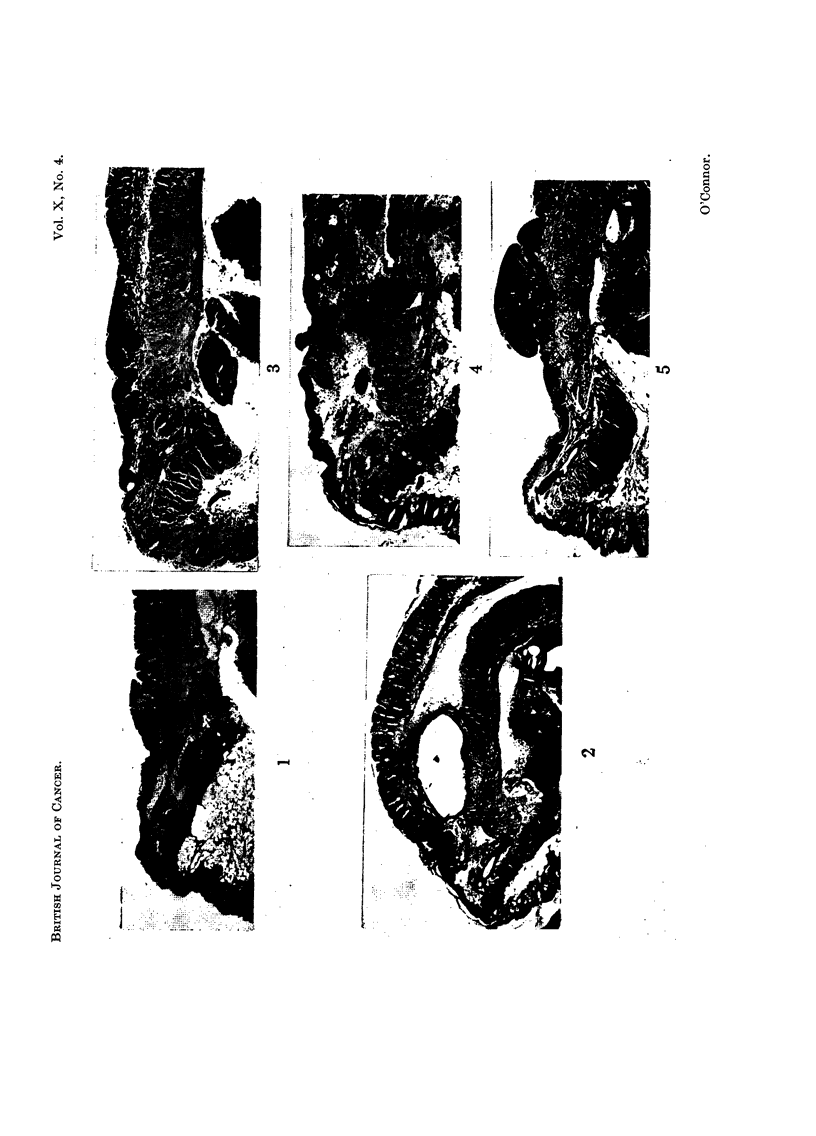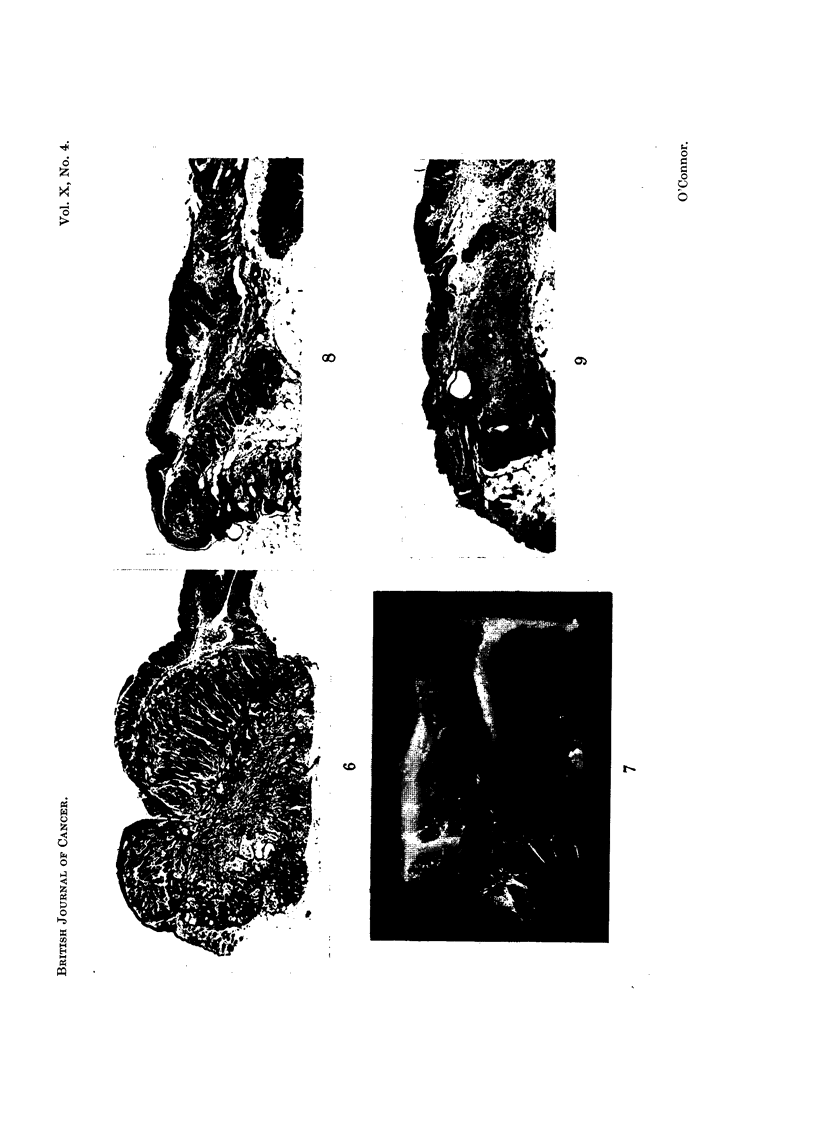# The Proliferative Capacity of the Lining Epithelium of the Anal Canal in Mice

**DOI:** 10.1038/bjc.1956.89

**Published:** 1956-12

**Authors:** R. J. O'Connor

## Abstract

**Images:**


### THE PROLIFERATIVE CAPACITY OF THE LINING EPITHELIUM OF THE ANAL CANAL IN MICE

R. J. O'CONNOR

*From the Westminster School of Medicine Research Laboratories, London, S.W.1*

Received for publication October 4, 1956

In this communication the results are recorded of implanting pellets of paraffin wax, containing methylcholanthrene, beneath the muco-cutaneous junction of the anal canal in mice. Since these experiments were designed to study the carcinogenic effect of methylcholanthrene on the mucous and squamous epithelium entering into the junction, results have been compared with those of similarly implanting control pellets containing no methylcholanthrene. In addition, the proliferation of the epithelium adjacent to the muco-cutaneous junction has been studied after the resection of part of the area of the anal lining in which the junction lies.

### MATERIALS AND METHODS

The mice used were from inbred laboratory stock but were of no particular strain. Fully-grown adults of both sexes were used but no differences due to sex were observed.

### *The implantation of paraffin pellets*

The pellets used were made of paraffin wax, melting point 56° C., and were about 2 mm. in diameter and less than 1 mm. thick. Methylcholanthrene, when present, was in a concentration of 5 per cent. The pellets were implanted into animals that had been anaesthetised with ether and then tied down on their backs when, by suitable retraction at the anal verge, the dorsal wall of the anal canal was exposed up to and beyond the muco-cutaneous junction. A small incision was then made at the anal verge, the pellet insinuated beneath the squamous epithelium of the anal canal, and pushed cranially until it lay beneath the muco-cutaneous junction and thus beneath both squamous and mucous epithelium. Retention of the pellet in this position was aided by closing the wound at the anal verge with a fine silk stitch. Subsequently, at various intervals, the effect on the anal lining of these pellets, with and without methylcholanthrene, was compared by making longitudinal sections of the anal canal.

### *Excision of the anal lining in the region of the muco-cutaneous junction*

As before, the dorsal wall of the anal canal was exposed in anaesthetised mice and from this aspect an area of the lining resected. The area extended 1–2 mm., both cranially and caudally, from the muco-cutaneous junction and about one-third of the circumference of the anal lining was removed. Care was taken to preserve intact the underlying muscular structures but some damage to them could not always be avoided. The consequences of such lesions were subsequently studied in longitudinal sections.

### OBSERVATIONS

### *The normal anatomy of the anal canal*

Many experiments resulted in an extension of the squamous epithelium cranial to the normal muco-cutaneous junction and thus into regions normally covered by mucous glands. This extension, although easily identified macroscopically, could only be assessed quantitatively in longitudinal sections by relating the muco-cutaneous junction to underlying structures. In the normal anal canal this junction lies at the level of the cranial border of an external sphincter of striated muscle which is separated from other muscle elements of the anal wall by the anal sebaceous glands and associated hair follicles. The transition from squamous to mucous epithelium is abrupt. Further, it should be noted that between the orifices of the anal glands and the muco-cutaneous junction, the squamous epithelium is devoid of both hair follicles and sebaceous glands (Fig. 1).

### *Following implantation of paraffin pellets*

To study the effect of methylcholanthrene on the anal lining changes were compared in experiments when the implanted pellets contained this substance and when they did not. Such comparisons were possible under two sets of circumstances ; firstly when the pellets remained in position beneath the muco-cutaneous junction, and, secondly, when, after remaining in this position for an arbitrarily chosen minimum period of 21 days, the pellets were discharged.

*When the pellets were retained.*—No significant differences were found when the pellets contained 5 per cent methylcholanthrene and when they did not. The effects found in both experiments are seen in Fig. 2, which illustrates an experiment in which a methylcholanthrene-containing pellet was implanted 83 days previously. The pellet lies beneath both the squamous and mucous epithelium at the muco-cutaneous junction. The mucous epithelium, apart from some stretching, shows no significant change and certainly none to suggest any abnormal proliferation. The significance of this negative finding is, however, considerably diminished for it was not possible to place the pellet superficial to the muscularis mucosae which therefore separates the methylcholanthrene in the pellet from the dividing cells in the bases of the mucous glands. On the other hand, in the vicinity of the pellets, changes are found in the squamous epithelium, but they are essentially the same whether or not it contains methylcholanthrene. These changes are also shown in Fig. 2. Immediately adjacent to the muco-cutaneous junction the squamous epithelium has become thickened with some downgrowth of epithelium into the underlying fibrous tissue but the downgrowth goes no deeper than this and does not reach the underlying muscle layers. Around the pellet is the tissue reaction often found around foreign bodies containing paraffin (cf. Gabriel, Dukes and Bussey, 1951), and this occurs to an equal extent when the pellet contains methylcholanthrene and when it does not. It can be added that such a reaction may shield adjacent cells from the action of methylcholanthrene and thereby account, in part, for the lack of carcinogenic effect both on the mucous and the squamous epithelium. In addition, no evidence of neoplastic transformation could be observed in the connective tissue cells in the neighbourhood of those pellets which contained methylcholanthrene.

*When pellets were extruded* 21 *days or more after implantation.*—Such extrusion took place through the mucous membrane immediately cranial to the muco-cutaneous junction. A breach was therefore created in the mucous membrane which became filled by a cranial extension of the squamous part of the lining of the anal canal. Superficially the magnitude of this extension could be determined by macroscopic examination of the lining of the anal canal after its removal. Thereby it was found that, neither in magnitude nor in frequency did this extension differ whether or not the extruded pellet had contained methylcholanthrene. In each case it occurred in about 75 per cent of experiments. In Fig. 3 such a cranial extension of squamous epithelium is shown in longitudinal section ; it can be seen to pass beyond the normal level of the muco-cutaneous junction at the cranial border of the anal glands and also of the external sphincter of striated muscle. The figure also demonstrates that, in addition to this superficial cranial extension, the squamous epithelium undergoes other proliferations. These additional proliferations, which are regularly found whether the extruded pellet contained methylcholanthrene or not, may take a variety of forms and, in this respect alone, do results with methylcholanthrene pellets differ from controls. The additional proliferations have therefore been arbitrarily classified into a number of groups and the frequency with which each occurs has been determined in experiments examined 80–180 days after the implantation of paraffin pellets at the muco-cutaneous junction and their subsequent extrusion. The groups chosen were as follows :

(*a*) Downgrowth of epithelium into underlying connective tissue but not deeper (Fig. 3).

(*b*) Downgrowth of squamous epithelium penetrating the muscle coats of the anal canal (Fig. 4).

(*c*) Papillomatous formations on the surface of the cranial extension of the squamous epithelium (Fig. 5).

(*d*) The formation of macroscopic tumours (Fig. 6). This figure shows the cranio-caudal extent of the largest of such tumours observed which, in this case, extended around the anal canal for more than half its circumference. In such tumours it was necessary to consider the possibility of malignant change but this appears to be unlikely since metastases were not found. Also, tumours of this type were well encapsulated and the component strands of squamous epithelium were regular in outline and in no way anaplastic (Fig. 6).

In Table I the frequency with which proliferations, so classified, were found is compared when the paraffin pellets contained methylcholanthrene and when they did not.

*Following resection of the anal lining at the muco-cutaneous junction*

Such resections, performed as already described, result in an extension cranially of squamous epithelium into the area normally covered by mucous glands. Since such extensions were found to be comparable with those following the extrusion of implanted paraffin pellets, they have been studied to determine the extent to which they, also, are associated with additional proliferations of the sort listed in Table I.

TABLE I.—*Proliferation of Squamous Epithelium at the Muco-cutaneous Junction Following the Implantation Beneath it of Paraffin Pellets and their Subsequent Extrusion After a Minimum Period of* 21 *Days*

| | Pellets containing methylcholanthrene | | Pellets without methylcholanthrene | |
|---|---|---|---|---|
| Group | No. | Per cent | No. | Per cent |
| *a* | 44 | 67 | 21 | 75 |
| *b* | 8 | 12 | 7 | 25 |
| *c* | 8 | 12 | — | — |
| *d* | 6 | 9 | — | — |
| Total | 66 | | 28 | |

The proliferations recorded were observed after 80–180 days, and, in every case, were additional to a superficial cranial extension of the squamous epithelium which occurred, to an equal extent when the pellets contained 5 per cent methylcholanthrene and when they did not. The groups *a*, *b*, *c*, *d*, into which the proliferations have been classified, are described in the text.

Within a week of performing the resection the reaction can be seen to differ in the persisting edges of the squamous and mucous epithelium. Proliferative changes in the latter are slight and confined to a scanty outgrowth, over the denuded area, of a single layer of epithelium. No further extension of the mucous membrane occurs and the area from which this membrane was resected becomes progressively covered by a cranial extension of squamous epithelium. This process becomes complete in 20–30 days (Fig. 7) and at the new junction of squamous and mucous epithelium the transition is as abrupt as in the normal. The early stages of this extension of squamous epithelium can be seen by the end of the second week, when in addition, there is a downgrowth of squamous epithelium into underlying tissue (Fig. 8). Such downgrowths were found, at this stage, in more than half of the resections performed. In a number of cases such additional proliferations persist after the cranial extension of squamous epithelium meets the persisting mucous membrane. Among these, in 2 of 20 specimens examined 100–150 days after the resection, the epithelial strands had penetrated into the muscle layers (Fig. 9) producing appearances similar to those of group *b*, (Table I and above) which were observed after the extrusion of implanted paraffin pellets not only when they contained methylcholanthrene but also when they did not.

### DISCUSSION

The results of resecting areas of anal lining containing the muco-cutaneous junction demonstrate so great a difference in the proliferative capacity of the squamous and mucous epithelium that the defect in the lining of the anal canal becomes completely covered by the former. This is in accordance with results previously recorded (O'Connor, 1954, 1956) when localised areas of mucosa were destroyed or resected in more proximal parts of the rectum and colon. No capacity of the mucosa to extend over denuded areas could be demonstrated. It seems reasonable to conclude therefore that the topographical stability of the muco-cutaneous junction depends on restraint of the proliferative capacity of the squamous epithelium due to its contact with the mucous. Evidence of such a restraint, may be seen in the abrupt transition, from squamous to mucous epithelium, which occurs both at the normal muco-cutaneous junction and at junctions experimentally produced at abnormal levels.

It is possible therefore to suggest, that if this contact is weakened by mechanical means, an excess of squamous proliferative capacity may take place at the muco-cutaneous junction. Such a weakening may occur as the result of the tension due to paraffin pellets implanted at the junction, and such is the explanation offered for the proliferation of squamous epithelium found when implanted pellets remain *in situ* (cf. Fig. 2). Such an explantion accounts, in addition, for the similarity of proliferation when, under such circumstances, the pellets contain methylcholanthrene and when they do not.

When the implanted pellets are subsequently extruded, a breach in the mucous membrane at the muco-cutaneous junction can be assumed to occur, into which squamous epithelium extends, independently of the presence or absence of methylcholanthrene in the implanted and extruded pellets. It is more doubtful however, whether the qualitative differences noted in the additional proliferations associated with this extension, recorded in Table I, are to be ascribed to the carcinogenic action of methylcholanthrene when it is present in the pellets. In the first place, the choice of the arbitrary and crude divisions into which the types of proliferation have been divided makes the data unsuitable for statistical analysis so that tests of the significance of the difference cannot be applied. Secondly, groups *a* and *b* not only occur when pellets contain no methylcholanthrene but they also occur, in a comparable form, following resection of areas of the anal lining which include the muco-cutaneous junction. Thirdly, neither groups *c* nor *d*, which were recorded only when the pellets contained methylcholanthrene, can, by conventional histological standards, be called malignant. Finally, the proliferations listed in Table I occur in squamous epithelium which has no hair follicles. Since the important role of these in chemical carcinogenesis has been stressed by Wolbach (1951), Whitely and Ghadially (1952) and by Liang and Cowdry (1955), it may be considered unlikely that methylcholanthrene exerts a carcinogenic effect when applied to the squamous epithelium of the anal canal.

In the case of the mucous epithelium at the anal muco-cutaneous junction, no evidence has been found to warrant any consideration of the possibility that methylcholanthrene applied as described, has a carcinogenic effect. Although, as already pointed out, in the present experiments, the contact between the epithelium and methylcholanthrene may be inadequate for carcinogenesis, these negative results correspond to the considerable amount of evidence collected by Laurens and Bacon (1952) showing that cells of mucous membrane of the large intestine are refractory to chemical carcinogens, and also to the findings that, under natural conditions, neoplasms in mice are rare in this region (Wells, Slye and Holmes, 1938).

### SUMMARY

Pellets of paraffin wax containing methylcholanthrene have been implanted beneath the muco-cutaneous junction of the anal canal of mice.

No effect, attributable to the carcinogen, could be demonstrated on the mucous epithelium entering into the junction.

Observations were made on a number of experiments in which, after remaining *in situ* for 3 weeks or more, pellets were extruded. Under such circumstances, proliferation of the squamous epithelium was more varied and extensive when the pellets contained methylcholanthrene than with control pellets. It was not possible however to establish that these differences were statistically significant.

These proliferations have been compared with those that follow resection of areas of the anal lining containing the muco-cutaneous junction. Under both circumstances the proliferations were sufficiently similar to make impossible the certain exclusion that all proliferations of squamous epithelium which follow the implantation of methylcholanthrene in paraffin pellets at the muco-cutaneous junction are non-specific in origin.

The difference in proliferative capacity of the squamous and mucous epithelium at the muco-cutaneous junction is so great that the topographical stability of the junction is ascribed to a restraint of proliferation exerted on the squamous epithelium by its contact with the mucous.

This work was performed while receiving a personal grant from the British Empire Cancer Campaign. Gratitude is expressed to Miss E. R. Blake for invaluable technical assistance.

### REFERENCES

GABRIEL, W. B., DUKES, C. E. AND BUSSEY, H. J. R.—(1951) *Brit. J. Surg.*, **38**, 401.
LAURENS, M. D. AND BACON, H. E.—(1952) *J. nat. Cancer Inst.*, **12**, 1237.
LIANG, H. M. AND COWDRY, E. V.—(1955) *Ibid.*, **16**, 205.
O'CONNOR, R. J.—(1954) *Brit. J. exp. Path.*, **35**, 545.—(1956) *Brit. J. Surg.*, **183**, 93.
WELLS, H. G., SLYE, M. AND HOLMES, H. F.—(1938) *Amer. J. Cancer*, **33**, 223.
WHITELY, H. J. AND GHADIALLY, F. N.—(1952) *J. Path. Bact.*, **64**, 651.
WOLBACH, S. B.—(1951) *Ann. N.Y. Acad. Sci.*, **53**, 517.

---

### EXPLANATION OF PLATES

FIG. 1.—Longitudinal section of normal anal canal showing relationship of the muco-cutaneous junction to the anal glands and the external sphincter of striated muscle. × 27.

FIG. 2.—Anal canal 83 days after the implantation of a paraffin pellet containing methylcholanthrene. Note the proliferation of squamous epithelium at muco-cutaneous junction. × 18.

FIG. 3.—Anal canal 141 days after the implantation at the muco-cutaneous junction and subsequent extrusion of a paraffin pellet containing methylcholanthrene. The squamous epithelium has extended cranially and there is also some proliferation into the underlying connective tissue. × 33.

FIG. 4.—Anal canal 126 days after the implantation at the muco-cutaneous junction and the subsequent extrusion of a paraffin pellet containing no methylcholanthrene. Proliferation of squamous epithelium with penetration into the underlying muscle coats. × 22.

FIG. 5.—Anal canal 148 days after the implantation at the muco-cutaneous junction and the subsequent extrusion of a paraffin pellet containing methylcholanthrene. Papillomatous proliferation of the squamous epithelium. × 22.

FIG. 6.—Anal canal 150 days after the implantation at the muco-cutaneous junction and the subsequent extrusion of a paraffin pellet containing methylcholanthrene. Development of a large tumour of squamous epithelium. The tumour is well encapsulated and there is no anaplasia of the constituent epithelial strands. × 14.

FIG. 7.—Anal canal 34 days after the resection of an area of its lining containing the muco-cutaneous junction. Cranial growth of the squamous epithelium into the area from which the mucous epithelium was removed. Specimen fixed in formalin : anal verge to the left. × 8.

FIG. 8.—Anal canal 10 days after the resection of an area of its lining containing the muco-cutaneous junction. Extension cranially of squamous epithelium with proliferation extending into the underlying connective tissue. × 31.

FIG. 9.—Anal canal 203 days after the resection of an area of its lining containing the muco-cutaneous junction. Extension cranially of the squamous epithelium with penetration into the underlying muscle coats. × 25